# Reverse epidemiology of elevated blood pressure among chronic hemodialysis black patients with stroke: a historical cohort study

**DOI:** 10.1186/s12882-017-0697-0

**Published:** 2017-08-30

**Authors:** Yannick Nlandu, François Lepira, Jean-Robert Makulo, Yannick Engole, Ernest Sumaili, Marie-Noelle Wameso, Vieux Mokoli, Jeannine Luse, Augustin Longo, Chantal Zinga, Pierre Akilimali, Aliocha Nkodila, Mélanie Bavassa, François Kajingulu, Justine Bukabau, Nazaire Nseka

**Affiliations:** 1 Nephrology Unit, Department of Internal medicine, Faculty of Medicine, University of Kinshasa Hospital, University of Kinshasa, BP 123 Kinshasa, Democratic Republic of Congo; 2 Nephrology Unit, Department of Internal medicine, General Provincial of Kinshasa Hospital, Kinshasa, Democratic Republic of Congo; 3 Kinshasa School of Public Health, University of Kinshasa Hospital, University of Kinshasa, Kinshasa, Democratic Republic of Congo; 4 Radiology Unit, Department of Internal medicine, Faculty of Medicine, University of Kinshasa Hospital, University of Kinshasa, Kinshasa, Democratic Republic of Congo

**Keywords:** Stroke, Hemodialysis, Incidence, Reverse epidemiology, Black people

## Abstract

**Background:**

Stroke is the third leading cause of cardiovascular mortality in dialysis patients. The objective of this study was to assess the extent of stroke in chronic hemodialysis patients.

**Methods:**

Historical cohort of patients enrolled in two hemodialysis (HD) centers from January 1, 2010 to December 31, 2011, including 191 patients (mean age 52 years, 68% men). Incidence curves and survival time analysis between the first day of HD and the end of the study were described by the Kaplan-Meier method. Independent stroke predictors were identified by multiple logistic regression analysis. *P* < 0.05 defined the level of statistical significance.

**Results:**

12 incident stroke were recorded during the study period, with 1622.1 person-months (PM), a stroke incidence rate of 7.4 cases per 1000 PM (95% CI = 7.35–7.44) at the point date. The incidence of stroke at 6 months, 12 months and 24 months was 9.8%, 11.9% and 13%, respectively. Only the absence of arterial hypertension (RR = 5.7, 95% CI: 1.52–21.42) emerged as an independent determinant of stroke.

**Conclusion:**

The high incidence of stroke in Kinshasa HD centers is partially explained by reverse epidemiology. Efforts must be made to understand this phenomenon in order to reduce its impact.

## Background

Cardiovascular disease (CVD) is the leading cause of death in maintenance Hemodialysis (HD), accounting for 40%–50% of all-cause mortality [1]. Stroke is the leading cause of cardiovascular mortality in patients with End Stage Renal Disease (ESRD) [1, 2]. Previous studies from developed countries reported 2- to 10-fold increased risk of stroke in dialysis patients compared to the general population [1]. This propensity for stroke has been attributed to the coexistence of traditional risk factors, such as hypertension and diabetes mellitus and those specifics to ESRD and dialysis, such as accelerated calcify arteriosclerosis, effects of uremic toxins, dialysis techniques, vascular access, and use of anticoagulants to maintain flow in the extracorporeal circuit [3, 4]. However, the relative contribution of these factors to the increased cardiovascular risk of dialysis patients remains a matter of debate [5, 6]. While some studies report a greater contribution of traditional risk factors [3, 5, 7], others underscore a more important role of factors specific to uremic or HD technique [5, 6, 8] or the conjunction of traditional and dialysis specific risk factors [5, 6, 9]. Hence, it seems rationale for each setting offering maintenance HD to evaluate the burden of stroke and the relative contribution of associated risk factors to inform policy makers and care providers.

Democratic Republic of the Congo (DRC), like other sub-Saharan African countries is experiencing an ever growing prevalence of non-communicable disease among which chronic kidney disease and end-stage renal disease [10]. Since 2007, HD centers mainly located in Kinshasa, the DRC capital city are providing maintenance HD. However, the burden of stroke and associated risk factors has not yet been evaluated. Therefore, we designed the present historical cohort study to estimate the incidence rates of stroke in patients undergoing HD and identify associated risk factors.

## Methods

Patients were recruited from two dialysis centers (Ngaliema Medical Center’s Hospital and General Hospital of Kinshasa) between January 1, 2010 and December 31, 2013. All patients aged ≥18 years old who received maintenance HD therapy for more than 3 months were included in the study. The follow-up period for each patient covered from their HD start date until its termination due to death, transplant and shift to peritoneal dialysis, transfer to another center or closing of the study. This is a historical cohort study. Baseline demographic data include age, gender, 24-h urine output, primary cause of ESRD, lifestyle habits (tobacco, alcohol), history of diabetes, hypertension, obesity, Hyperuricemia, hypercholesterolemia or CVD, and biochemical parameters, including hemoglobin, serum albumin, serum calcium, serum phosphorus, total cholesterol, serum C-reactive protein (CRP), serum uric acid, serum urea nitrogen, serum creatinine, and dialysis dose as KT/ V. These data were obtained at the start of HD. All parameters were measured in the central laboratory of both hospitals. The study major outcome was stroke, which is defined as a focal neurological deficit of cerebrovascular persisting for >24 h diagnosed as an ischemic or hemorrhagic stroke by computed tomography (CT). The diabetes diagnosis at the initiation of dialysis was based on diagnostic criteria from the American Diabetes Association [11]. Hypertension was recorded if the patient was taking any antihypertensive drug or had two separate blood pressure measurements ≥140/90 mmHg. Obesity was defined by a BMI ≥ 30 Kg/m^2^. Hyperuricemia and hypercholesterolemia was recorded respectively if the patient had an acid uric serum ≥7 mg/dl and a total cholesterol serum ≥200 mg/dl. Smoking was defined by a consumption of at least 1 cigarette / day for 5 years or more (active smoking) or withdrawal for less than 5 years (past smoking). Alcoholism was defined by a consumption of at least 20 g of alcohol / day or >2 glasses of beer / day for at least one year. The presence of pre-dialysis comorbid conditions, including history of stroke was determined by a review of medical chart. Cardiovascular Disease (CVD) retrieved from medical chart included left ventricular hypertrophy (LVH), myocardial infarction, atherosclerotic heart disease, cardiomyopathy, cardiac arrhythmia, cardiac arrest, congestive heart failure, cerebrovascular accident, ischemic and peripheral vascular disease. Body mass index (BMI) was calculated as weight (kg) divided by height (m) squared. The pulsed pressure (PP) was calculated as follows: PP = SBP-DBP. The mean arterial pressure (MAP) as follows: MAP = DBP + (SBP - DBP) / 3. KT/V was measured by an Online Clearance Monitor (OCM) (Fresenius Healthcare Ltd). The phosphocalcic product was calculated by the product between phosphoremia (mg / dl) and serum calcium also (mg / dl).

Summary statistics were presented as percentages for categorical data, mean ± standard deviation for approximately normally distributed continuous variables, and median for skewed continuous variables. According to stroke events, patients were also divided into two groups: stroke and non-stroke. Characteristic differences between two groups were tested by using the chi-square test for categorical variables, Student’s t-test for approximately normally distributed continuous variables, and the non-parametric Mann-Whitney test for skewed continuous variables. The cumulative incidence of stroke was determined using the Kaplan Meier method. Variables found to be significant were used in logistic analysis model using an SAS package. The dependent binary variable in this model was presence (1) or absence (0) of stroke. Statistical significance was defined as *p* < 0.05. Statistical analyses were performed using SPSS 17.0 for Windows.

The study protocol was approved by the Clinical Research Ethics Committee of Public Health’s School at the University of Kinshasa (Kinshasa, DRC). Since this is a retrospective study, written consent was not required.

## Results

**Fig. 1:**
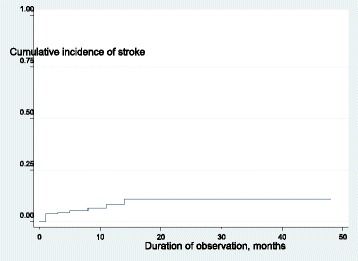
cumulative incidence of stroke curve calculated by Kaplan –Meier method

**Fig. 2:**
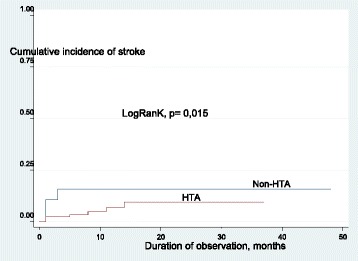
Cumulative incidence of stroke curve in HTA and non HTA group by Kaplan-Meier method

Characteristics of the patients at the start of the study are shown in Table 1. A total of 191 patients were registered (129 males and 62 females, sex ratio 2.1) and the median period of follow up on HD was 5.8 months. The mean age of patients was 52.3 ± 12.3 years ranging from 15 to 76 years. It was 54.0 ± 12.2 years in male and 48.7 ± 11.9 years in female. The mean SBP of patients was 155.7 ± 27.6 mmHg. The main cardiovascular risk factors were anemia (93.7%), hypertension (85.3%), LHV (44.1%) and diabetes mellitus (41,4%). The percentage of patients taking aspirin, statin and various types of anti-hypertensive agents was respectively 28.3, 34.2 and 85.3%. The primary cause of ESRD was Diabetes (64 patients, 33.5%) followed by Glomerular disease (54 patients, 28.3) and Hypertension (48 patients, 25.1%). In 122 patients (63.9%) dialysis was done 2 times per week and in 49 patients (42.6%) KT/V was inferior to 1.3. During the four-year study period, we recorded 12 cases (only men) of stroke with intra-cerebral hemorrhage (ICH) and ischemic subtypes in 4 (33,3%) and 8 (66,7%) patients, respectively. The cumulative incidence of stroke increased linearly only in the first year and reaching approximatively 13% after 4 years. CRP levels were higher in the presence of stroke than in the absence [6.5 (5.0–12.0) vs. 6.3 (4.1–12.0); *p* = 0.029]. Compared to patients without stroke, those with stroke had a significantly higher proportion of male subjects (100 vs. 65.4%; *p* = 0.013), Hyperuricemia (25 vs. 17.9%; *P* = 0.038), LVH (63.6 vs. 42.8%, *p* = 0.015) and prior stroke history (25 vs. 17.9; *p* = 0.038); however, they had a significantly lower proportion of Hypertension (66.7% vs. 86.6%, *p* = 0.008). SBP, PP and MAP were in average lower in the presence of stroke. The results of multiple logistic analyses are shown in Table 2. Absence of Hypertension was identified as a significant predictor of stroke (OR 5.7; 95%CI 1.52–21.42). The incidence of stroke in the whole group as well as in hypertensive and non-hypertensive patients was 7.4, 0.55 and 2.31 per 1000 patients-months, respectively (Figs. 1 and 2).

**Table 1:** Comparative baseline characteristics of patients with and without stroke

Variables	Non Stroke *n* = 179	Stroke *n* = 12	p
Demographics			
Age ≥ 55 years, n(%)	89 (49,7)	6 (50,0)	0,985
Male, n(%)	117 (65,4)	12 (100,0)	0,013
Duration of HD (month)	5,4 (3–37)	6,2 (3–48)	0,560
Body mass index (Kg/m^2^)	24,8 ± 4,8	22,9 ± 3,5	0,318
Etiologies of renal disease			
• Diabetic nephropathy	61 (34,1)	3 (25.0)	0,519
• Chronic glomerulonephritis	53 (29.6)	1 (8.3)	0.113
• Hypertensive	44 (24.6)	4 (33.3)	0.499
• HIVAN	7 (3.9)	1 (8.3)	0.044
• Polycistic Kidney Disease	3 (1.7)	0	0.651
• Obstructive uropathy	9 (5.0)	1 (8.3)	0.619
• Chronic pyelonephritis	2 (1.1)	1 (8.3)	0.052
Comorbidities			
• Cardiovascular diseases (CVD)	89 (49.7)	7 (58.3)	0.391
• History of cancer	10 (5.6)	2 (16.3)	0.017
• HIV	9 (5.0)	2 (16.7)	0.015
• Rhumatismal disease	17 (9.5)	2 (16.7)	0.340
Laboratory variable			
Serum creatinine (mg/dl)	13,9 ± 8,8	14,3 ± 5,9	0,901
GFR, ml/min/1.73 m^2^	6,06 ± 3,3	5,17 ± 2,3	0,363
Serum urea nitrogen (mg/dl)	219,7 ± 10,2	269,2 ± 12,2	0,105
Serum uric acid (mg/dl)	8,0 ± 2,9	8,87 ± 3,	0,460
Calcemia (mEq/l)	4,3 ± 0,7	4,1 ± 0,6	0,272
Serum phosphorus (mg/dl)	5,0 ± 2,3	4,6 ± 1,6	0,692
CaxP (mg^2^/dl^2^)	44,1 ± 19,4	34,4 ± 16,8	0,335
Total cholesterol (mg/dl)	187,8 ± 63,4	164,2 ± 56,2	0,551
Serum albumin (g/l)	37,6 ± 8,4	37,4 ± 5,9	0,227
Total protein (g/l)	65,5 ± 12,2	68,3 ± 9,8	0,550
Hemoglobin(g/dl)	8,1 ± 1,9	8,5 ± 2,1	0,519
CRP (mg/l)	6,3 (4,1–12,0)	6,5 (5,0–12,0)	0,029
24 h–proteinuria (g)	1,7 (1,2–2,2)	1,5 (1,3–2,3)	0,999
Risk factors for CVD			
Hypertension, n(%)	155 (86,6)	8 (66,7)	0,008
Systolic blood pressure (mm Hg)	156,9 ± 27,7	136,8 ± 19,0	0,014
Diastolic blood pressure(mm Hg)	86,8 ± 18,9	79,2 ± 17,0	0,174
Mean arterial pressure (mm Hg)	110,0 ± 19,8	98,4 ± 16,3	0,044
Pulse Pressure (mm Hg)	70,5 ± 21,2	56,8 ± 15,1	0,029
Diabetes Melitus, n(%)	76 (42,5)	3 (25,0)	0,189
Obesity, n(%)	32 (17,9)	4 (33,3)	0,169
Prior stroke, n(%)	32 (17,9)	3 (25,0)	0,038
Anemia, n(%)	168 (93,9)	11 (91,7)	0,552
LVH, n(%)	71 (42,8)	7 (63,6)	0,015
Hyperuricemia, n(%)	32 (17,9)	3 (25,0)	0,038
Hypercholesterolemia, n(%)	44 (24,6)	2 (16,7)	0,414
Tabac, n(%)	15 (8,4)	0 (0,0)	0,363
Alcohol, n(%)	34 (19,0)	4 (33,3)	0,197
KT/V			
< 1,3	46 (43.0)	3 (37.5)	0.533
≥ 1.3	61 (57.0)	5 (62.5)

**Table 2:** Multiple logistic analysis of predictors of stroke in chronic hemodialysis population

Predictors	n	person-months	Number of stroke	Incidence of stroke per 100 P-M	OR unadjusted (IC95%)	p	OR adjusted (IC95%)	p
Hypertension, n(%)								
- No	28	61	4	6,56	4,39 (1,30–14,87)	0,017	6,11 (1,69–21,99)	0,006
- yes	163	441	8	1,81	1		1	
LVH								
- No	99	278	4	1,44	1		1	
- Yes	78	187	7	3,74	2,97 (0,86–10,24)	0,085	3,43 (0,96–12,32)	0,058
Prior Stroke								
- No	156	405	9	2,22	1		1	
- Yes	35	97	3	3,09	1,29 (0,35–4,79)	0,707	0,81 (0,17–3,94)	0,794

## Discussion

The incidence of stroke in this study was 7.4 per 1000 patient-months. This observation corroborates data from the literature reporting a higher incidence of stroke in chronic hemodialysis compared to the general population [3–8]. Indeed, the incidence of stroke observed in this study is higher than that of 1.123 per 1000 patient-years reported in a meta-analysis of some available African studies [12]. This incidence is also higher than that of 0.8 per 1000 patient-years in African hospital settings and those in chronic hemodialysis patients in Europe and Asia who varied between 17 and 74 per 1000 patient-years [3–8]. The propensity of chronic hemodialysis patients to develop more strokes relies mainly upon the acceleration of the dynamic process of atherosclerosis already initiated by uremia and its underlying cause [13]. Indeed, hemodialysis accelerates, through oxidative stress and subsequent inflammation, pre-existing remodeling of all vascular beds especially cerebral vessels [13, 14]. The particular vulnerability of brain vessels to the deleterious effects of uremia and hemodialysis is related to the structural and functional similarities between renal glomerular microcirculation and cerebral microcirculation [15, 16]. Indeed, the large extra-cerebral arterial trunks (aorta and carotids) play an important role of shock absorber (“Windkessel effect”) to modulate hemodynamic stress from the heart to the brain [15, 16]. Similar to renal glomerular microcirculation, the peculiar nature of cerebral microcirculation, characterized by high blood flow and low vascular resistance, makes the brain vulnerable to pulsatory hemodynamic stress during each cardiac cycle [15, 16]. With the acceleration of atherosclerosis through uremia and hemodialysis, the wall of the central elastic arteries (aorta and carotid arteries) loses its elasticity and becomes rigid, resulting in loss of shock function and direct transmission from hemodynamic stress to cerebral microcirculation [15, 16]. This will result in structural and functional lesions responsible for a wide range of subclinical and clinical conditions including stroke [15, 16]. Apart from these structural similarities, the high incidence of stroke in chronic hemodialysis patients can also be explained by several other factors including the coexistence of uremic (anemia, hyperphosphorémie, ADMA retention and NO inhibitor) and traditional risk factors as well as the passive selection by hemodialysis of an elderly population with multiple comorbidities [4, 6].

In the present study, non-hypertensive patients are at increased risk of stroke suggesting a paradoxical protective effect of elevated blood pressure in chronic hemodialysis. This observation disagrees with the high risk of stroke conferred by elevated blood pressure in the general population. Indeed, INTERSTROKE Study shows that hypertension is the main risk factor for all stroke subtypes, particularly among young Africans who develop stroke while ignoring their hypertensive status [17]. The apparent paradoxical protection afforded by elevated blood pressure against stroke observed in the present study can be partly explained by the phenomenon of reverse epidemiology of traditional risk factors reported both in ESRD and chronic dialysis [18, 19]. This inverse relationship does not necessarily mean that the principles of vascular physiopathology of atherosclerosis are different in patients with ESRD under dialysis; It seems rather to indicate the existence of additional and more powerful risk factors such as inflammation and malnutrition which apparently alter the relationship between traditional risk factors and outcomes in dialysis patients [18, 19]. This phenomenon, not specific to chronic kidney disease and dialysis, is also reported in chronic conditions such as chronic heart failure, cancer and chronic infections like HIV/AIDS [18, 19]. Another plausible explanation of the apparent paradox can be systolic cardiac dysfunction [18, 19]. Indeed, hypertension-induced cardiac failure is associated with low blood pressure or hypotension that is both a marker of cardiac disease severity and a powerful predictor of morbidity and mortality [18, 19]. Low blood pressure in uremic patients can also be a reflection of autonomic neuropathy that, in turn, is a marker for more severe uremic complications [18, 19]. Apart from the aforementioned mechanisms underlying reverse epidemiology of hypertension, more recent studies have suggested this phenomenon to be mainly due to inadequacy of peridialytic BP recordings per se to describe the true BP load, rather than a true U-shaped relationship of BP with cardiovascular morbidity and mortality [20]. Indeed, peridialytic BP recordings are not made for diagnostic reasons but to exploit a major hemodynamic metric like BP in order to assess cardiovascular stability before, during and immediately after the dialysis procedure. Thus, using these readings to diagnose hypertension, assess the success of antihypertensive treatment or examine future cardiovascular risk could be inherently hazardous [20].In contrast to the unclear association of peridialytic BP recordings with all-cause and cardiovascular mortality, recent prospective studies have shown that interdialytic BP recorded either at home or by ambulatory BP monitoring (ABPM) associates more closely with mortality and cardiovascular events, as it is documented for the general population [20]. In practice, these data suggest that there is probably no benefit to recommended achieving normal BP in dialysis. Reverse epidemiology has misleading relevance on dialysis management. The high early mortality universally associated with low baseline BP figures does not contradict the need to achieve normal BP in dialysis patients to reduce long-term cardiovascular mortality. Besides, the eventual noxious/beneficial role of antihypertensive medications in dialysis patients needs to be investigated.

### Limitations

Our study has some limitations that must be acknowledged. First, our results cannot be generalized to all hemodialysis patients since it is not a multicentric study. Second, the study design based on a “post-hoc analysis” of the data for another purpose does not identify the different characteristics (duration, severity ...) of the stroke which was not the main target of the study. Third, the small sample size does not give enough power to statistical tests to identify potential associations between variables of interest.

## Conclusion

Incidence of stroke is higher in Congolese patients receiving hemodialysis with non-hypertensive patients having the highest risk suggesting the reverse epidemiology of elevated blood pressure in chronic hemodialysis.

## Abbreviations

BMI: Body Mass Index
CRP: C Reactive Protein
CT: Computed tomography
CVD: Cardiovascular Disease
DBP: Diastolic Blood Pressure
DRC: Democratic republic of Congo
ESRD: End Stage Renal Disease
HD: Hemodialysis
ICH: Intra-Cerebral hemorrhage
LVH: Left Ventricular hypertrophy
MAP: Mean Arterial Pressure
OCM: Online Clearance Monitor
SAS: Statistical Analysis Software
SBP: Systolic Blood Pressure
